# Dietary Fatty Acid Regulation of the NLRP3 Inflammasome via the TLR4/NF-*κ*B Signaling Pathway Affects Chondrocyte Pyroptosis

**DOI:** 10.1155/2022/3711371

**Published:** 2022-05-04

**Authors:** Xin Jin, Xin Dong, Yingxu Sun, Ziyu Liu, Li Liu, Hailun Gu

**Affiliations:** ^1^Department of Nutrition and Food Hygiene, School of Public Health, China Medical University, 110122, China; ^2^Department of Orthopedics, Shengjing Hospital, China Medical University, 110004, China

## Abstract

Dietary fatty acid (FA) content and type have different effects on obesity-associated osteoarthritis (OA), but the mechanisms underlying these differences are not fully understood. Inflammation activated by toll-like receptor 4 (TLR4)/nuclear factor- (NF-) *κ*B signaling and pyroptosis induced by the NLRP3/caspase-1/gasdermin D (GSDMD) signaling pathway play important roles in OA development. Our aim in this study was to observe the effects of dietary FAs on the articular cartilage of obese post-traumatic OA model mice and on chondrocytes stimulated by lipopolysaccharide (LPS) and to determine whether the underlying mechanisms involve TLR4/NF-*κ*B and NLRP3/caspase-1/GSDMD signaling pathways. Mice were fed high-fat diets rich in various FAs and underwent surgical destabilization of the medial meniscus to establish the obesity-related post-traumatic OA model. LPS-induced SW1353 chondrosarcoma cells were used to mimic OA status *in vitro*, and TLR4 inhibitors or TLR4 overexpressing lentivirus was administered. Analysis using weight-matched mice and multiple regression models revealed that OA was associated with dietary FA content and serum inflammatory factor levels, but not body weight. Diets rich in n-3 polyunsaturated fatty acids (PUFAs) attenuated OA and inhibited the TLR4/NF-*κ*B and NLRP3/caspase-1/GSDMD signaling pathways, whereas diets rich in saturated fatty acids (SFAs), monounsaturated fatty acids (MUFAs), or n-6 PUFAs increased OA severity and activated these pathways. *In vitro* results for SFAs, n-6 PUFAs, and n-3 PUFAs were consistent with the animal experiments. However, those for MUFAs were not. FA effects on the NLRP3/caspase-1/GSDMD pathway were associated with the inhibition or activation of the TLR4 signaling pathway. In conclusion, diets rich in SFAs or n-6 PUFAs can exacerbate obesity-associated OA, whereas those rich in n-3 PUFAs have protective effects against this disease, due to their respective pro-/anti-inflammatory and pyroptotic effects. Further research on dietary FA supplements as a potential therapeutic approach for OA is needed.

## 1. Introduction

Osteoarthritis (OA) is a degenerative joint disease that affects 237 million people worldwide and is an important cause of disability in middle-aged and older adults [1]. The joint changes of OA, including articular cartilage degeneration, subchondral bone thickening, bone fragment formation, synovitis, and the degeneration of ligaments and menisci, result in decreased function and quality of life. Patients with OA face significant healthcare and social costs [2–4]. Joint trauma, aging, metabolic disorders, and obesity can trigger OA [5], but the pathogenesis of this disease is still not well known and needs to be investigated further.

In recent years, with the gradual increase in the prevalence of obesity, the incidence of obesity-related OA has increased [6]. In addition to obesity-related biomechanical risk factors, which increase intra-cartilage pressure and aggravate wear and tear in joints [7], obesity-related chronic inflammation and nutritional metabolism abnormalities play roles in the development of OA [8, 9]. Excessive nutritional intake leads to lipid influx beyond the storage capacity of adipose tissues, resulting in elevated circulatory fatty acid (FA) levels [10]. Increased FA accumulation in joints has been shown to be associated with cartilage damage severity in OA, with different FA types having distinct effects [11, 12]. Saturated fatty acids (SFAs) can increase the release of inflammatory factors such as interleukin- (IL-) 1*β*, causing degenerative changes in chondrocytes and having adverse effects on OA onset and progression [13]. Studies examining the effects of monounsaturated fatty acids (MUFAs) on OA have yielded variable results [14]. n-3 polyunsaturated fatty acids (PUFAs) have long been considered to be anti-inflammatory FAs with protective effects against OA [15]. However, inconsistent with previous results, the n-3 PUFA docosahexaenoic acid (DHA) was recently reported to induce pyroptosis in microglia and breast cancer cells [16, 17]. Conversely, n-6 PUFAs are considered to be pro-inflammatory FAs that increase cartilage degradation while promoting synovial inflammation, thereby accelerating OA progression [18]. Cohort studies have also shown that systemic levels of n-6 PUFAs correlate positively with the OA risk [19] and that increased ratios of n-6 to n-3 PUFAs have deleterious effects on cartilage [20]. However, the mechanisms mediating these different effects are not fully understood.

Toll-like receptor (TLR) 4, a member of the TLR family, mediates innate and adaptive immune responses [21, 22]. Through its downstream molecule myeloid differentiation factor (MyD)88, it signals to activate the nuclear factor- (NF-) *κ*B pathway, leading to the production of various inflammatory cytokines [23]. TLR4 plays an important role in the development of OA [24].

Pyroptosis is a type of programmed cell death that is distinct from apoptosis and characterized by the disruption of the structural integrity of the cell membrane, leading to the release of cellular contents and thus the activation of a strong inflammatory response. Pyroptosis is mediated by inflammasomes, the most widely studied and well-characterized type of which is the NOD-like receptor protein (NLRP) 3 inflammasome [25]. NLRP3 inflammasome–mediated pyroptosis depends on the caspase-1–mediated classical inflammasome pathway and the caspase-4, -5, and -11–mediated non-classical inflammasome pathway. These pathways lead to the cleavage of the gasdermin D (GSDMD) protein, which releases the gasdermin-N structural domain, resulting in cell membrane pore formation, inflammatory factor release, and, ultimately, cell death [26–29]. Activated NLRP3 and caspase-1 effects depend on K^+^ ion efflux, reactive oxygen species production, and phagocytosis to produce IL-1*β*, IL-18, and matrix-degrading enzymes, which exacerbates the pyroptosis inflammatory response, leading to cartilage degeneration and synovitis, and thereby accelerated OA progression [30, 31].

The differences in FA effects on OA may be due to differences in their mechanisms of regulating TLR4 and pyroptosis. SFAs can activate TLR4, producing an inflammatory response [32]. They can also increase the expression of the NLRP3 inflammasome, the activated form of which mediates the cleavage of pro–caspase-1 into active caspase-1, which cleaves pro–IL-1*β* and pro–IL-18 into their mature forms. The mature IL-1*β* and IL-18 molecules are secreted through cell membrane pores constituted by GSDMD proteins and exacerbate the inflammatory response to pyroptosis [33–35]. In contrast, n-3 PUFAs can inhibit inflammatory signaling pathways by suppressing TLR-induced signaling [36, 37] and suppress NLRP3 inflammasome activity, resulting in anti-inflammatory effects and the inhibition of pyroptosis development [38]. Few studies have examined the effects of MUFAs and n-6 PUFAs on TLR4 signaling and pyroptosis, and their results have been mixed.

The link between the TLR4/NF-*κ*B signaling pathway and the NLRP3 inflammasome and its downstream signals may also be important for the understanding of FA effects on OA. In cardiomyocytes, SFAs can bind MD protein 2 directly, which induces MD2/TLR4 complex formation and the production of pro-inflammatory cytokines that activate TLR4 signaling, generating an inflammatory response [32] and activating NLRP3 inflammasomes. These inflammasomes, in turn, drive caspase-1 activation and GSDMD protein cleavage, ultimately leading to pyroptosis [39]. The effects of other FA types on inflammasomes remain unclear. Further exploration of FA effects on TLR4/NF-*κ*B signaling and NLRP3 inflammasome–induced pyroptosis and the relationship between the two signaling pathways, may help to elucidate the roles of different FA mechanisms in OA, revealing possible intervention targets for obesity-related OA.

Thus, in the present study, we examined the hypothesis that different types of FA promote/reduce OA by up/down-regulating TLR4/NF-*κ*B signaling, which in turn increases/decreases NLRP3 inflammasome expression to regulate inflammation and pyroptosis. To observe the effects of diets rich in different types of FA on obesity-associated OA, mice were fed high-fat diets rich in particular FAs (SFAs, MUFAs, n-6 PUFAs, or n-3 PUFAs) and subjected to surgical destabilization of the medial meniscus (DMM). To further examine the mechanisms of FA regulation of chondrocyte pyroptosis via TLR4 regulation, we tested the effects of different types of FA on the NLRP3 inflammasome and its downstream signals via activated or inhibited TLR4 expression in an *in-vitro* OA model produced by treating cells with lipopolysaccharide (LPS). Our study may provide an experimental basis for the elucidation of the mechanisms underlying the effects of different types of FA on OA and the identification of potential therapeutic targets.

## 2. Materials and Methods

### 2.1. Animal Handling and Surgery

Six-week-old male C57BL/6 mice (weighing 18–22 g, *n* = 75) were purchased from the Department of Laboratory Animals of China Medical University. All animal experiments were approved by the Professional Committee on Animal Use and Ethics of China Medical University (SCXKLN2013-0009). The mice were randomized to five groups (*n* = 15 each) according to their body weights after 1 week of acclimatization feeding and then fed a low-fat diet (LD) or a SFA-rich, MUFA-rich, n-6 PUFA-rich (n-6), or n-3 PUFA-rich (n-3) diet (feed formulations are provided in Table S1). All groups of mice were fed and watered freely and maintained at room temperature (20–25°C) with 40–70% relative humidity. Their body weights and food intakes were recorded weekly.

At week 18, all mice underwent surgery consisting of sham surgery on the left hind limb and DMM on the right hind limb. After the operations, group-designated feeding was continued until week 26, at which time gait analysis and body fat measurements were performed, blood was drawn from the abdominal vein under ether anesthesia, the mice were killed by cervical dislocation, and both hind limbs were measured.

### 2.2. Histology

Whole knee joints (*n* = 5) were fixed in 4% paraformaldehyde, decalcified, paraffin-embedded, and sectioned at 6 *μ*m thickness for histological and immunohistochemical evaluation. The sections were stained with saffron O (Solarbio, Beijing, China), hematoxylin-eosin (Solarbio), or saffron O/solid green (Solarbio) to reveal histological changes in the cartilage. Cartilage destruction severity was graded according to the modified Mankin [40] and Osteoarthritis Research Society International (OARSI) [41] scoring systems (scoring criteria are provided in Tables S2 and S3).

### 2.3. Gait Analysis

The automated Noldus (Wageningen, the Netherlands) system was used for gait analysis. Mice (*n* = 5) were placed individually in a CatWalk walkway and allowed to walk freely and traverse from one side to the other of the walkway's glass plate. Each mouse completed three successful walking tests in the test channel. Each footprint was imaged and analyzed using the CatWalk XT10.6 software (Noldus). The following parameters were calculated: print area (the complete surface area contacted by the paw during a stance phase), maximum contact (the total floor area contacted by the paw during the stance phase), duty cycle (stance duration as a percentage of the step cycle duration), and swing speed (speed of a paw's movement during the swing phase).

### 2.4. Immunohistochemistry

Antigen repair enzymes were added dropwise to slide-mounted sections, following the instructions of the immunohistochemistry kit used (MXB, Fujian, China). Then, the sections were incubated overnight with anti-NLRP3 (1 : 100; Abcam, Cambridge, UK), anti–caspase-1 (1 : 100; Abcam, Cambridge, UK), and anti–IL-1*β* (1 : 100; ImmunoWay, Plano, TX, USA) rabbit primary antibodies at 4°C. Thereafter, the sections were incubated with horseradish peroxidase- (HRP-) conjugated anti-rabbit immunoglobulin G (IgG; Abcam) for 1 h at room temperature and cover-slipped with neutral balsam. Six fields of view on each section were selected randomly for optical density (OD) analysis at 400x under a light microscope (Olympus, Tokyo, Japan) using ImageJ (NIH Image, Washington DC, USA).

### 2.5. Enzyme-Linked Immunosorbent Assays

Enzyme-linked immunosorbent assays (ELISAs) were conducted in 96-well plates with ELISA kits (Boster, Wuhan, China) in accordance with the manufacturer's instructions. Briefly, 100 *μ*L mouse serum samples (*n* = 5), 100 *μ*L biotin-labeled antibody, and 100 *μ*L ABC working solution were added to each plate well. Then, TMB color development solution was added dropwise. The color was allowed to develop in the dark for 15–20 min, and then 100 *μ*L TMB reaction termination solution was added. OD values were determined at 450 nm with a microplate reader.

### 2.6. Cell Culture

SW1353 cells were purchased from the Cell Resource Center of Shanghai Institutes for Biological Sciences, Chinese Academy of Sciences. They were cultured in Dulbecco's modified Eagle medium (DMEM; Gibco, Grand Island, NY, USA) containing 10% fetal bovine serum (FBS; BI, Beit-Haemek, Israel) and 1% penicillin-streptomycin (KeyGEN Bio, Jiangsu, China) in a humidified incubator at 37°C and 5% CO_2_.

### 2.7. Cell Treatment

#### 2.7.1. Cell Proliferation/Viability Assay

Logarithmic-growth-phase SW1353 cells were incubated in 96-well plates with complete medium combined with LPS (Sigma, St. Louis, MO, USA; 12.5, 25, 50, 100, 200, 500, 1000, or 2000 ng/mL). Palmitic acid (PA), oleic acid (OLA), linolenic acid (LA), or DHA (Sigma; 1, 6.25, 12.5, 25, 50, 100, or 200 *μ*M) was added, and the cells were then incubated at 37°C for 24 h. To assess cell concentrations, 10 *μ*L cell counting kit-8 reagent (KeyGEN Bio) was added to each well, and the plates were incubated in the dark for 2 h. Subsequently, OD values were measured at 450 nm using a microplate reader. Each assay was performed in triplicate.

#### 2.7.2. FA Treatment

Cells were treated with LPS (1000 ng/mL) only or LPS plus an FA (concentrations of three different gradients) for 24 h. They were then collected for analysis.

#### 2.7.3. CLI-095 Treatment

Cell media were replaced with DMEM containing 0.5% FBS and the TLR4 inhibitor CLI-095 (1 *μ*g/mL; InvivoGen, San Diego, CA, USA) for 6 h. LPS (1000 ng/mL) was added with or without PA or LA (25 *μ*M) and allowed to incubate for 24 h.

#### 2.7.4. Cell Transfection with TLR4 Overexpression Lentivirus

Cells were transfected with TLR4 overexpression or negative control (NC) lentivirus (GenePharma, Shanghai, China) in DMEM containing 0.5% FBS for 6 h and then cultured in DMEM containing 10% FBS for 72 h. Puromycin (2 *μ*g/mL) was added to screen for stably transfected cells. Following screening, transfected cells were exposed to media supplemented with LPS (1000 ng/mL), with or without OLA or DHA (25 *μ*M), for 24 h.

### 2.8. Western Blot

Articular cartilages (*n* = 5) were ground under liquid nitrogen. Chondrocytes (*n* = 3) were collected, and protein was extracted directly by adding RIPA lysis buffer (Sangon Biotech, Shanghai, China) on ice. Protein concentrations were determined with a bicinchoninic acid kit (Dingguo Bio, Beijing, China). Sodium dodecyl sulphate–polyacrylamide gel electrophoresis was conducted, and the resultant protein bands were transferred to polyvinylidene fluoride membranes for 3 h. The membranes were blocked over 30 min at room temperature and then incubated overnight at 4°C with primary antibody targeting collagen II (1 : 500; Bioss, Beijing, China), *β*-actin (1 : 500; Bioss, Beijing, China), NLRP3 (1 : 1000; Abcam, Cambridge, UK), GSDMD (1 : 1000; Abcam, Cambridge, UK), caspase-1 (1 : 1000; Abcam, Cambridge, UK), TLR4 (1 : 1000; Santa Cruz Biotechnology, Santa Cruz, CA, USA), MyD88 (1 : 1000; Santa Cruz Biotechnology, Santa Cruz, CA, USA), or IL-1*β* (1 : 500; ImmunoWay, Plano, TX, USA). Subsequently, the membranes were incubated with HRP-conjugated goat anti-rabbit secondary antibody IgG (1 : 5000; ImmunoWay, Plano, TX, USA) for 1 h at room temperature. Following exposure to electrochemiluminescence reagent, images were developed and grayscale values of the bands were determined with ImageJ (NIH Image, Washington DC, USA). Semi-quantitative analysis was performed with *β*-actin serving as an internal reference. All experiments were repeated three times.

### 2.9. Immunofluorescence

Cells were cultured in 24-well plates, fixed in 4% paraformaldehyde, and then treated with 0.5% Triton X-100. After blocking with bovine serum albumin (Dingguo Biotech), the cells were incubated overnight with anti-GSDMD and anti–caspase-1 primary antibodies (1 : 100; Abcam, Cambridge, UK) at 4°C. After rinsing with Tris-buffered saline with 0.1% Tween 20, the cells were incubated with dropwise-added fluorescence-labeled goat anti-rabbit IgG (1 : 200; Zhongshan Jinqiao, Beijing, China) at room temperature in the dark for 2 h. The slices were sealed with an anti-fluorescence quencher (Dingguo Bio) and incubated for 5 min in the dark before being imaged under an inverted fluorescence microscope. Six randomly selected fields of view in each examined section were subjected to OD analysis at 200x using ImageJ (NIH Image, Washington DC, USA).

### 2.10. Statistical Analyses

The results are expressed as means ± standard deviations. One-way analyses of variance were used for comparisons. When the data met the homogeneity of variance test condition, the least significant difference *t* test was used for two-way comparisons; otherwise, Dunnett's T3 method was used. In all cases, *p* < 0.05 was considered to reflect significant differences. The data were analyzed using SPSS 23.0 software (IBM Corporation, Armonk, NY, USA).

## 3. Results

### 3.1. Effects of Dietary FAs on Mouse Body Weight, Body Fat, and Food Intake

**Figure 1:**
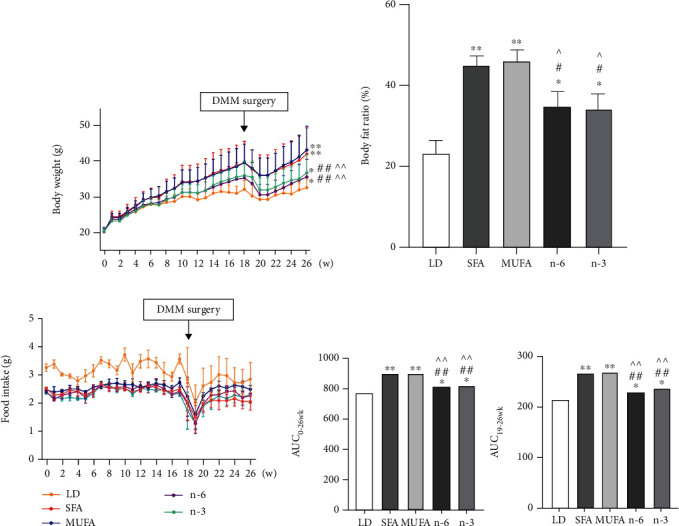
Effects of dietary FAs on body weight, body fat, and food intake in mice. (a) Body weight. (b) Body fat ratio at the end of 26 weeks of feeding. (c) Food intake. (d) The areas under weight curve of different FAs were calculated for the period from 0 to 26 weeks (AUC_0-26wk_) and from 19 to 26 weeks (AUC_19-26wk_), respectively. The data are presented as the means ± SD, *n* = 15. ^∗^*p* < 0.05 and ^∗∗^*p* < 0.01 versus the LD group; ^#^*p* < 0.05 and ^##^*p* < 0.01 versus the SFA group; ^*p* < 0.05 and ^^*p* < 0.01 versus the MUFA group.

After 26 weeks of dietary treatment, the body weights and body fat ratios of mice in all high-fat diet groups were significantly greater than those in the LD group (Figures 1(a) and 1(b)). These values were lower in the n-6 and n-3 PUFA groups than in the SFA and MUFA groups. Food intake did not differ among high-fat diet groups (Figure 1(c)). To accurately detect the effects of body weight change over time on joints, we calculated areas under the body weight curve (AUCs) for mice in the different diet groups from baseline to 26 weeks and 19 to 26 weeks, respectively. The 0–26- and 19–26-week AUCs were higher in the high-fat groups than in the LD group, and higher in the SFA and MUFA groups than in the n-6 and n-3 PUFA groups (Figure 1(d)).

### 3.2. Effects of Dietary FAs on Mouse Articular Cartilage

**Figure 2:**
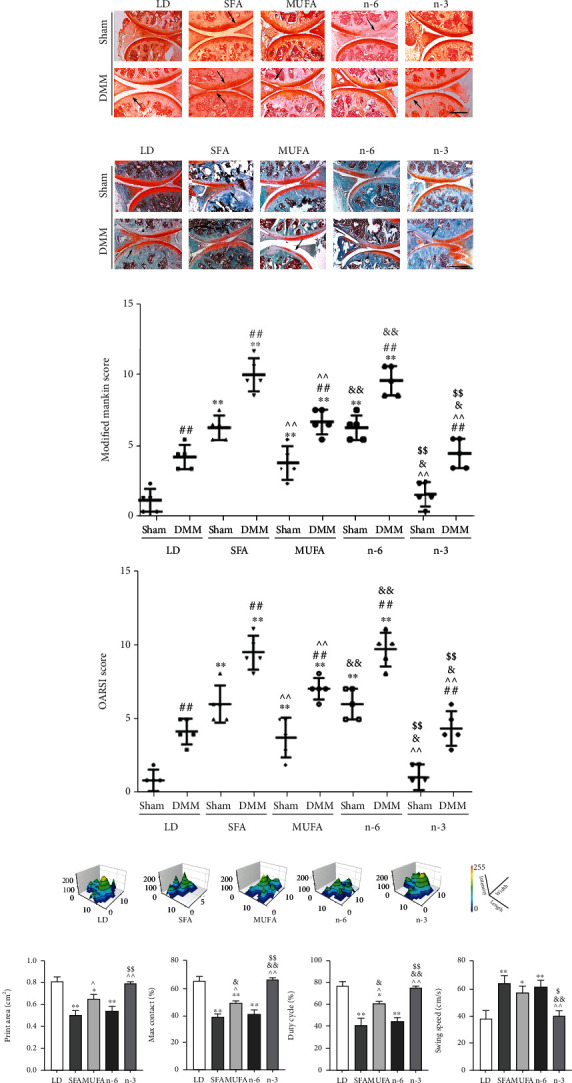
Effects of dietary FAs on articular cartilage in mice. (a) H&E staining (200x; scale bars = 100 *μ*m). The arrows represented local aggregation or absence of cartilages. (b) Safranin O staining (100x; scale bars = 200 *μ*m). The arrows indicated cartilage thinning or loss. (c) Safranin O/Fast Green staining (100x; scale bars = 200 *μ*m). The arrows indicated the fuzzy fracture of the tide line. (d) Modified Mankin scores and OARSI scores. (e) Histogram of footprints of mice in each group from DMM hind limbs. (f) The bar graph data of print area (cm^2^), max contact (%), duty cycle (%), and swing speed (cm/s). The data are presented as the means ± SD, *n* = 5. ^∗^*p* < 0.05 and ^∗∗^*p* < 0.01 versus the LD group; ^#^*p* < 0.05 and ^##^*p* < 0.01 versus the sham group; ^*p* < 0.05 and ^^*p* < 0.01 versus the SFA group; ^&^*p* < 0.05 and ^&&^*p* < 0.01 versus the MUFA group; ^$^*p* < 0.05 and ^$$^*p* < 0.01 versus the n-6 group.

Sham-operated joints from the SFA and n-6 PUFA groups showed mild OA-like damage, whereas those from the MUFA and n-3 PUFA groups showed no significant abnormality. The degree of OA in joints subjected to DMM was significantly augmented in all groups, but lesions in the n-3 PUFA group were similar to those in the LD group and less severe than those in the other groups (Figures 2(a)–2(c)). The Mankin and OARSI scores for joints subjected to DMM were significantly worse in the SFA and n-6 PUFA groups than in the LD and n-3 PUFA groups. Scores for the MUFA group were higher than those for the LD and n-3 PUFA groups, but lower than those for the SFA and n-6 PUFA groups (Figure 2(d)). Compared with the LD group, the SFA, MUFA, and n-6 PUFA groups had more irregular gaits; significantly smaller footprint area, maximum contact, duty cycle, swing speed, maximum footprint contact, and support ratio values; and significantly increased swing speeds in the DMM-modified limbs. The most significant differences were between the SFA and n-6 PUFA groups, and no significant difference was observed between the LD and n-3 PUFA groups (Figures 2(e) and 2(f)).

### 3.3. Relationships among Dietary FAs, Body Weight, Serum Inflammatory Factors, and OA in Mice

**Figure 3:**
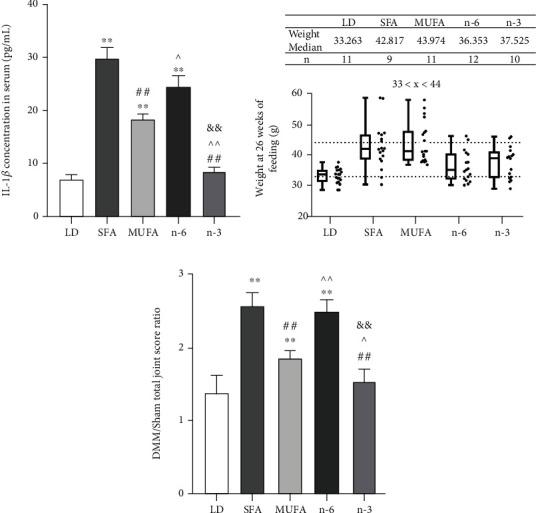
The expression of IL-1*β* in serum and weight-matched strategy for analyzing the relationship between diet FAs and OA severity. (a) The concentration of IL-1*β* in serum was detected by ELISA, *n* = 5. (b) The mice whose weights were in the range of 33 to 44 grams were used for OA analysis at 26 weeks of feeding. The line of the box indicated median, and the length of the box represented interquartile range. (c) DMM-operated to sham-operated joint OA score ratio of the weight-matched mice. The data are presented as the means ± SD. ^∗^*p* < 0.05 and ^∗∗^*p* < 0.01 versus the LD group; ^#^*p* < 0.05 and ^##^*p* < 0.01 versus the SFA group; ^*p* < 0.05 and ^^*p* < 0.01 versus the MUFA group; ^&^*p* < 0.05 and ^&&^*p* < 0.01 versus the n-6 group.

Serum IL-1*β* levels were significantly higher in the SFA, MUFA, and n-6 PUFA groups than in the LD group and did not differ between the LD and n-3 PUFA groups. The MUFA group's mean serum IL-1*β* level was significantly higher than that in the n-3 PUFA group and lower than that in the SFA and n-6 PUFA groups (Figure 3(a)). The DMM/sham joint score ratios were significantly higher in the SFA, n-6 PUFA, and MUFA groups than in the LD group after controlling for body weight factors (Figures 3(b) and 3(c)). Multiple regression analysis showed that dietary FAs and IL-1*β* levels were associated significantly with OA, rather than body weight (Table 1).

### 3.4. Effects of Dietary FAs on TLR4/NF-*κ*B Signals and the NLRP3 Inflammasome and Its Downstream Signals in Mouse Articular Cartilage

**Figure 4:**
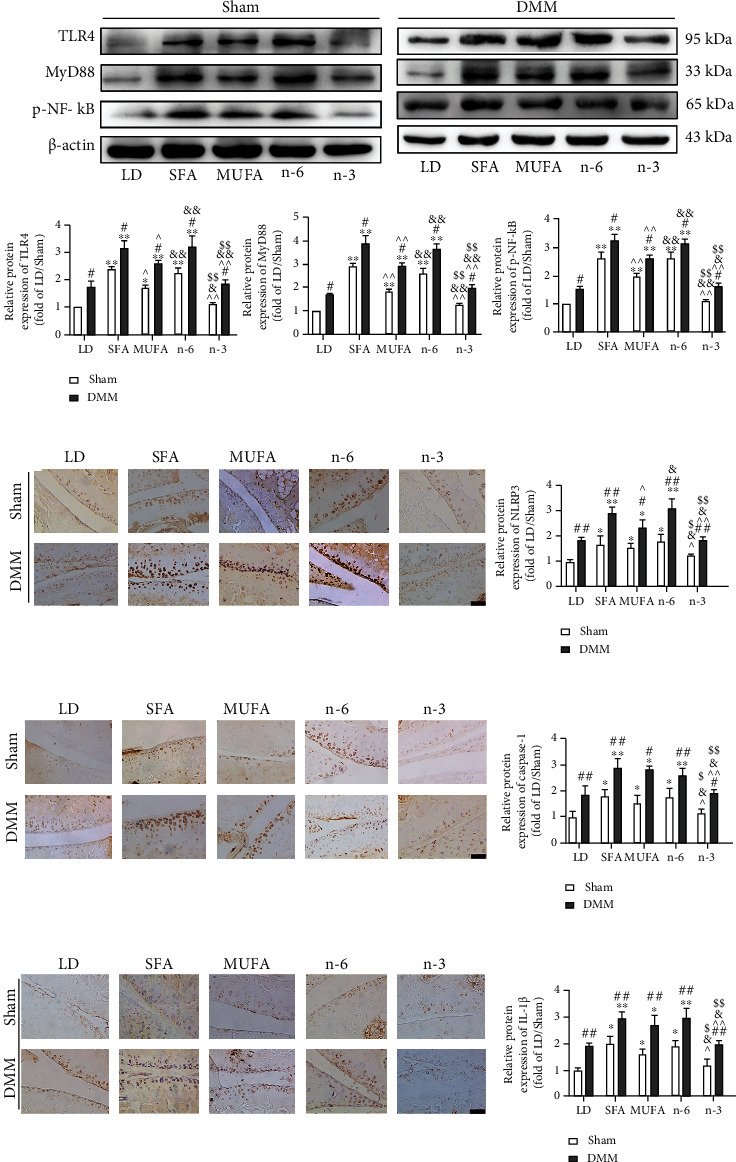
Effects of dietary FAs on TLR4/NF-*κ*B signals and NLRP3 inflammasome as well as its downstream signals in articular cartilage of mice. (a) The relative expression of TLR4, MyD88, and p-NF-*κ*B protein of knee articular cartilage was detected by Western blot. (b–d) Immunohistochemical staining was used to measure NLRP3, caspase-1, and IL-1*β* positive cells (200x, scale bars = 100 *μ*m). The data are presented as the means ± SD, *n* = 5. ^∗^*p* < 0.05 and ^∗∗^*p* < 0.01 versus the LD group; ^#^*p* < 0.05 and ^##^*p* < 0.01 versus the sham group; ^*p* < 0.05 and ^^*p* < 0.01 versus the SFA group; ^&^*p* < 0.05 and ^&&^*p* < 0.01 versus the MUFA group; ^$^*p* < 0.05 and ^$$^*p* < 0.01 versus the n-6 group.

Western blot analysis of articular cartilage from joints subjected to DMM and sham operation showed that TLR4, MyD88, and phosphorylated- (p-) NF-*κ*B protein levels were significantly higher in the SFA, MUFA, and n-6 PUFA groups than in the LD and n-3 PUFA groups and that those in the latter two groups were similar. These levels were significantly higher in the SFA and n-6 PUFA groups than in the MUFA group. For all groups, TLR4, MyD88, and p-NF-*κ*B protein levels were higher in articular cartilage from joints subjected to DMM than in that from joints subjected to sham operation (Figure 4(a)).

Similarly, immunohistochemical analysis showed significantly increased NLRP3, caspase-1, and IL-1*β* expression in the SFA, MUFA, and n-6 PUFA groups compared with the LD and n-3 PUFA groups, with no significant difference between the latter. For all groups, the expression levels of these proteins were significantly higher in cartilage from joints subjected to DMM than in sham operation joints (Figures 4(b)–4(d)).

### 3.5. Effects of Different FA Types on Chondrocyte Viability and Proliferation

**Figure 5:**
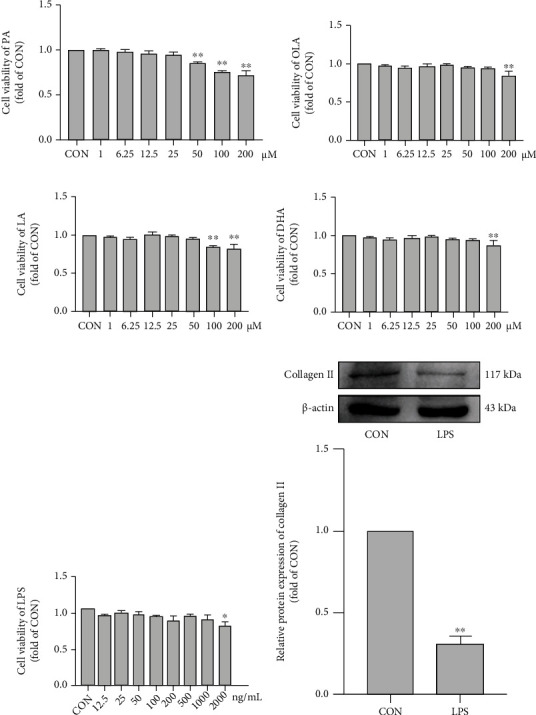
Effects of different FA types on chondrocyte viability and proliferation. Cell viability of PA (a), OLA (b), LA (c), and DHA (d) on SW1353 cells. (e) Cell viability of LPS on SW1353 cells. (f) The relative protein expression of Collagen II in SW1353 cells treated with LPS (1000 ng/mL) was detected by Western blot analysis. The data are presented as the means ± SD, *n* = 3. ^∗^*p* < 0.05 and ^∗∗^*p* < 0.01 versus the CON group.

PA, OLA, LA, and DHA did not affect the viability or proliferation of SW1353 chondrocytes at concentrations ≤ 25 *μ*M. PA at the concentration of 50 *μ*M, LA at the concentration of 100 *μ*M, and OLA and DHA at the concentration of 200 *μ*M reduced cell viability and proliferation (Figures 5(a)–5(d)). At concentrations ≤ 1000 ng/mL, LPS had no significant effect on the viability or proliferation of cells (Figure 5(e)). However, 1000 ng/mL LPS significantly reduced type II collagen expression in the cells (Figure 5(f)). Thus, we used 1000 ng/mL LPS to treat cells to mimic OA status *in vitro*.

### 3.6. SFA and n-6 PUFA Treatments Enhanced TLR4/NF-*κ*B Signaling and the NLRP3/caspase-1/GSDMD Signaling Pathway, whereas n-3 PUFA and MUFA Treatments Attenuated Them, in Chondrocytes

**Figure 6:**
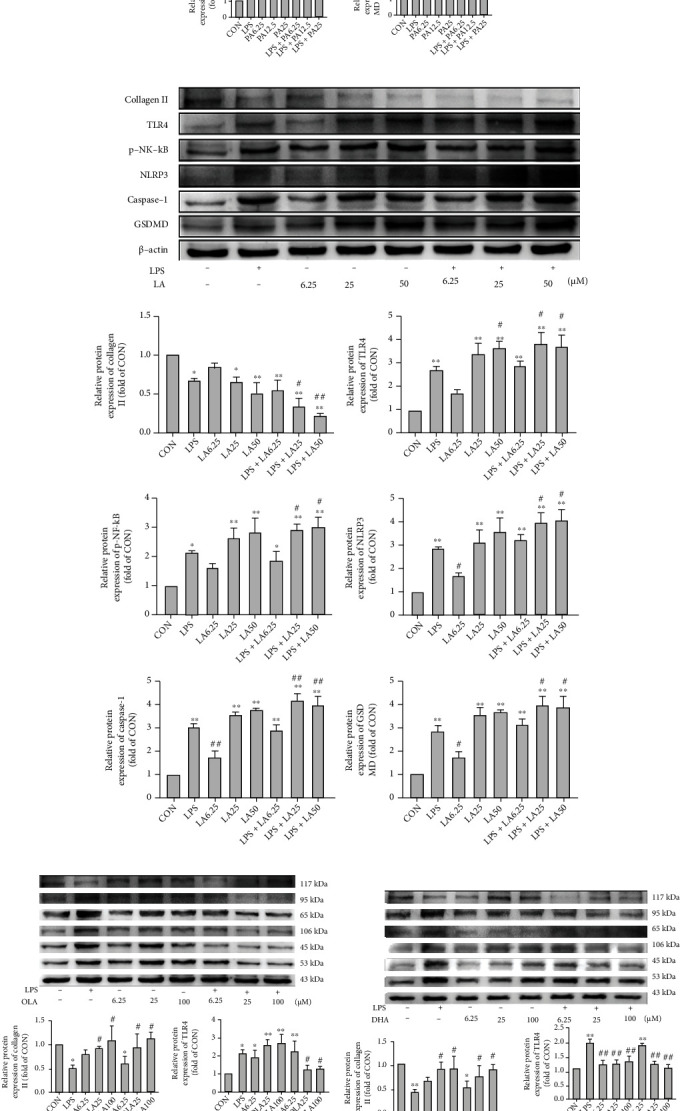
Effects of FAs on TLR4/NF-*κ*B signaling and NLRP3/caspase-1/GSDMD signaling in chondrocytes. The relative protein expression of collagen II, TLR4, p-NF-*κ*B, NLRP3, caspase-1, and GSDMD in SW1353 cells treated with various concentrations of PA (a), LA (b), OLA (c), and DHA (d) in the absence or presence of LPS for 24 h. The data are presented as the means ± SD, *n* = 3. ^∗^*p* < 0.05 and ^∗∗^*p* < 0.01 versus the CON group; ^#^*p* < 0.05 and ^##^*p* < 0.01 versus the LPS group.

LPS treatment significantly increased the expression of TLR4, p–NF-*κ*B, NLRP3, and the pyroptosis-related proteins caspase-1 and GSDMD. PA and LA at the concentration of 25 *μ*M reduced type II collagen expression and increased TLR4, p-NF-*κ*B, NLRP3, caspase-1, and GSDMD expression. These treatments also enhanced the LPS induction effects (Figures 6(a) and 6(b)). In contrast, cells exposed to 25 *μ*M concentrations of OLA and DHA showed a significant increase in type II collagen and dramatic decreases in TLR4, p-NF-*κ*B, NLRP3, caspase-1, and GSDMD levels and could reverse LPS-induced changes in these proteins (Figures 6(c) and 6(d)).

### 3.7. TLR4 Inhibition Attenuated the SFA- and n-6 PUFA–Mediated Activation of NLRP3/caspase-1/GSDMD Signaling

**Figure 7:**
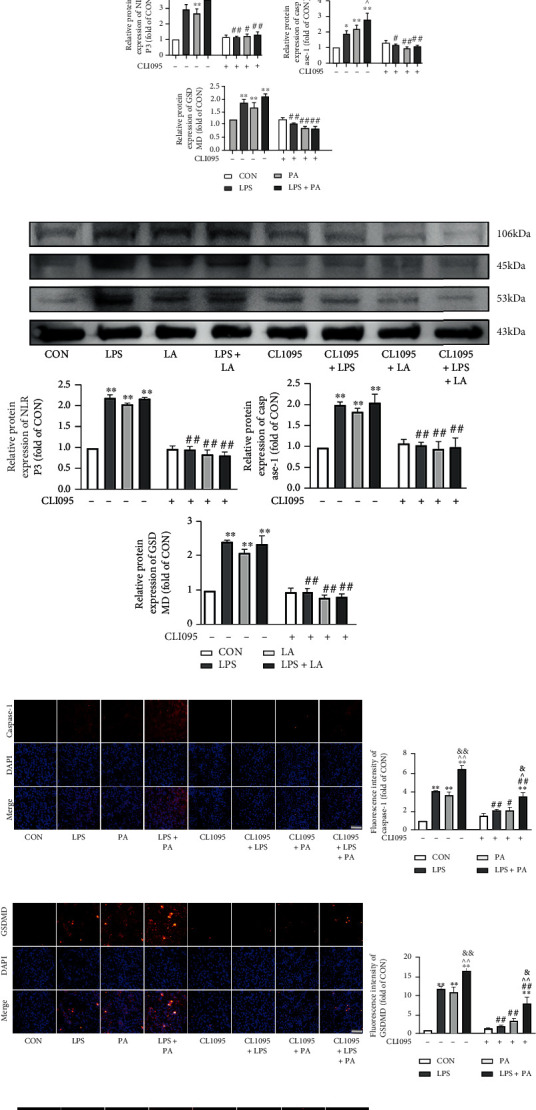
TLR4 inhibition attenuated SFA- and n-6 PUFA-mediated activation of NLRP3/caspase-1/GSDMD signaling. The relative protein expression of collagen II, TLR4, p-NF-*κ*B, and IL-1*β* in SW1353 cells treated with PA (a) or LA (b). The relative protein expression of NLRP3, caspase-1, and GSDMD in SW1353 cells treated with PA (c) or LA (d). Immunofluorescent staining was used to detect the expression of caspase-1 (e, g) and GSDMD (f, h) in SW1353 cells treated with PA or LA (200x, scale bars = 100 *μ*m). The data are presented as the means ± SD, *n* = 3. ^∗^*p* < 0.05 and ^∗∗^*p* < 0.01 versus the CON group; ^#^*p* < 0.05 and ^##^*p* < 0.01 versus the groups without CLI-095-pretreatment; ^*p* < 0.05 and ^^*p* < 0.01 versus the LPS group; ^&^*p* < 0.05 and ^&&^*p* < 0.01 versus the PA or LA group.

TLR4 inhibition with CLI-095 increased chondrocyte type II collagen expression significantly while decreasing TLR4, p-NF-*κ*B, NLRP3, caspase-1, GSDMD, and IL-1*β* protein expression (Figures 7(a)–7(d)). Additionally, TLR4 inhibitor treatment reversed the decrease in type II collagen and upregulation of p-NF-*κ*B, NLRP3, caspase-1, GSDMD, and IL-1*β* protein expression induced by PA and LA (Figures 7(a)–7(d)). caspase-1 and GSDMD immunofluorescence analyses yielded similar results (Figures 7(e)–7(h)).

### 3.8. TLR4 Overexpression Reversed the Inhibitory Effects of n-3 PUFA and MUFA on NLRP3/caspase-1/GSDMD Signaling in Chondrocytes

**Figure 8:**
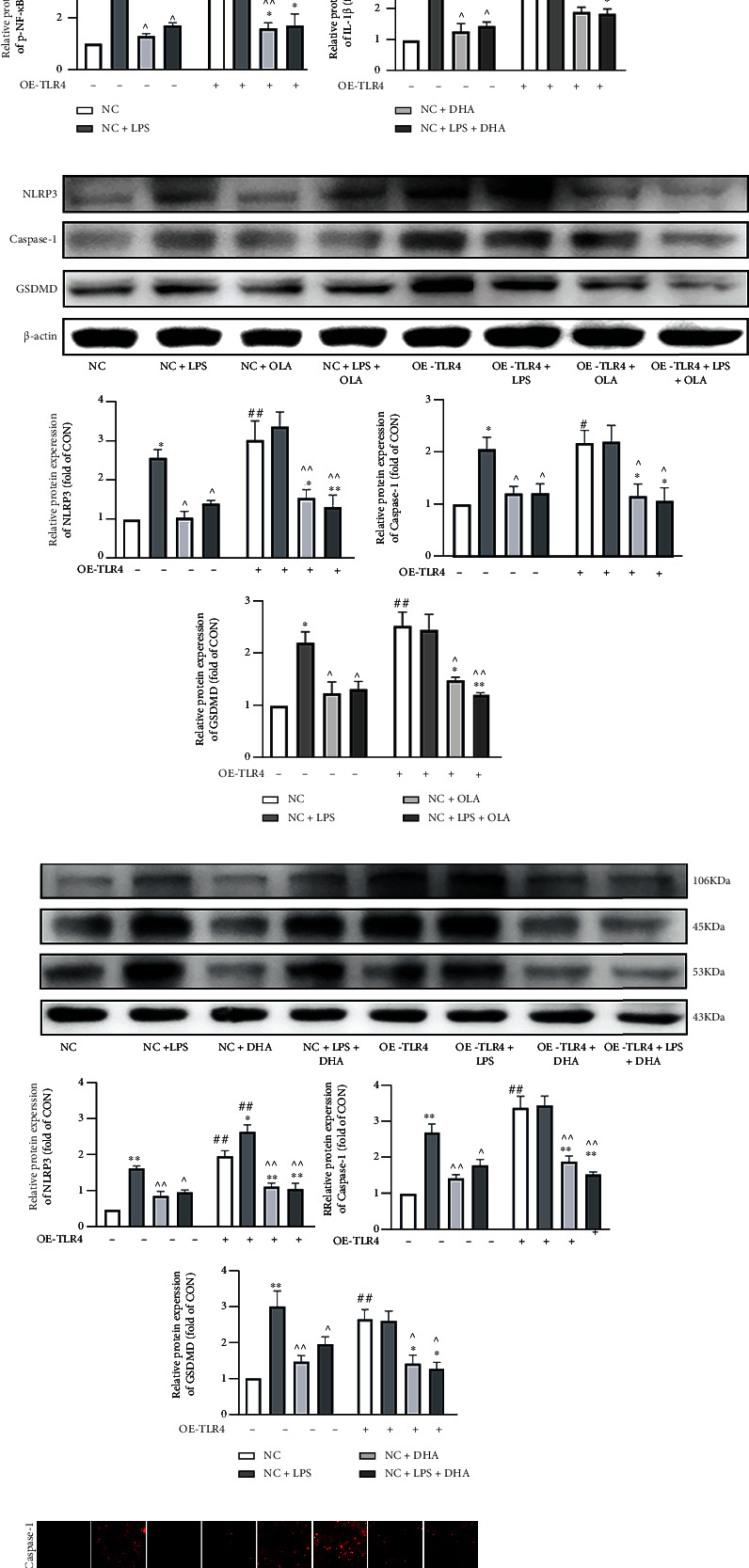
TLR4 overexpression reversed the inhibitory effect of n-3 PUFA and MUFA on NLRP3/caspase-1/GSDMD signaling in chondrocytes. The relative protein expression of collagen II, TLR4, p-NF-*κ*B, and IL-1*β* in SW1353 cells treated with OLA (a) or DHA (b). The relative protein expression of NLRP3, caspase-1, and GSDMD in SW1353 cells treated with OLA (c) or DHA (d). Immunofluorescent staining was used to detect the expression of caspase-1 (e, g) and GSDMD (f, h) in SW1353 cells treated with OLA or DHA (200x, scale bars = 100 *μ*m). The data are presented as the means ± SD, *n* = 3. ^∗^*p* < 0.05 and ^∗∗^*p* < 0.01 versus the NC group; ^#^*p* < 0.05 and ^##^*p* < 0.01 versus the groups without OE-TLR4-pretreatment; ^*p* < 0.05 and ^^*p* < 0.01 versus the NC+LPS group; ^&^*p* < 0.05 and ^&&^*p* < 0.01 versus the NC+OLA or NC+DHA group.

We used 30 multiplicity-of-infection TLR4 overexpression lentivirus in combination with puromycin to treat and screen chondrocytes (Fig. S1). Chondrocytes transfected with TLR4 overexpression lentivirus had significantly reduced type II collagen expression and markedly increased TLR4, p-NF-*κ*B, NLRP3, caspase-1, GSDMD, and IL-1*β* protein expression compared with the NC group (Figures 8(a)–8(d)). Additionally, TLR4 overexpression lentivirus treatment reversed the increase in type II collagen and down-regulation of p–NF-*κ*B, NLRP3, caspase-1, GSDMD, and IL-1*β* protein expression induced by OLA and DHA (Figures 8(a)–8(d)). This pattern of caspase-1 and GSDMD results was replicated in the immunofluorescence experiments (Figures 8(e)–8(h)).

## 4. Discussion

Obesity is a major risk factor for the development of OA, which is associated with a chronic low-grade inflammatory state and elevated circulating levels of adipokines and free FAs. Diet composition, especially FA content and caloric intake, may impact OA severity in a joint-specific manner [42, 43]. Notably, n-6/n-3 PUFA dietary ratios have been associated with markers of metabolic disorders, including obesity, insulin resistance, and inflammation. SFA, MUFA, and n-3 and n-6 PUFA have been detected in the synovial fluid of knee joints of patients with and without OA, and the n-6/n-3 PUFA ratio is significantly higher in patients with OA [44].

In the present study, the n-6/n-3 PUFA ratio in the feed for the n-6 PUFA group was close to that of the traditional Western diet (i.e., 24.6 : 1). Conversely, the n-6/n-3 PUFA ratio in the feed given to the n-3 group was the generally recommended healthy ratio of 4.2 : 1, and the ratio for the SFA and MUFA groups matched that of the LD group (7.4 : 1). Compared with mice fed a low-fat diet, mice fed diets rich in SFAs or n-6 PUFAs showed mild OA-like changes in articular cartilage, and the lesions in joints subjected to DMM surgery were more severe. Consistent with our findings, Wu et al. [18] found that mice fed SFA- and n-6 PUFA–enriched diets tended to develop severe OA and synovitis with systemic inflammation. In the present study, the joints of mice fed diets rich in MUFAs or n-3 PUFAs that were not subjected to DMM had no significant lesions. Joints in the MUFA group changed significantly after DMM surgery, and mice in the n-3 PUFA group developed lesions to a similar extent as did those in the LD group (much less than in the SFA, MUFA, and n-6 PUFA groups). These results suggest that n-3 PUFAs have a protective effect on the development of obesity-related OA and contribute to posttraumatic joint repair.

Because the mean body weights of mice fed high-fat diets were significantly greater than those of the LD group and those of mice in the SFA and MUFA groups were greater than those of mice in the n-6 and n-3 PUFA groups, we controlled for the confounding factor of body weight [18]. After such control, we found that the above-mentioned differences among groups persisted, further indicating that the anti-OA effect of n-3 PUFA was independent of body weight and supporting the inference that the intergroup differences can be attributed to the effects of different types of FA. Diets rich in n-3 PUFAs appear to attenuate traumatic OA in obese mice and support the repair of OA-like damage, whereas the consumption of SFAs and n-6 PUFAs has been associated with greater osteophyte formation, synovitis, and macrophage infiltration of the synovium [9].

Little is known about the effects of MUFAs on joints. In this study, our histological and gait analyses revealed the effects of a high-fat diet rich in MUFAs on mouse articular cartilage were similar to those of a diet rich in n-3 PUFAs. However, MUFAs did not reduce cartilage damage caused by DMM. This negative finding, combined with the serum IL-1*β* level data, suggests that a diet rich in MUFAs also has a proinflammatory effect, but to a much less extent than do diets rich in SFAs or n-6 PUFAs. Our results suggest that dietary factors play a more important role in obesity-related posttraumatic OA than do mechanical factors. Different types of FA may have different effects on OA, and exploring their mechanisms of action is of great value for the prevention and treatment of obesity-related OA from a dietary perspective.

TLR4 is the most expressed TLR in chondrocytes, and its expression in joint tissues increases with OA severity [45]. Moreover, the inhibition of TLR4 expression in cartilage has been shown to reduce OA severity in rats [46]. The TLR4 signaling pathway is considered to be a main trigger of obesity-induced inflammation, and the TLR4/NF-*κ*B signaling pathway is an important mechanism that regulates the chondrocyte OA inflammatory response [47]. In addition, obesity disrupts mitochondrial integrity, leading to the release of reactive oxygen species, which triggers the formation of NLRP3 inflammasome complexes [48]. Activation of the NLRP3 inflammasome activates caspase-1, mediates cellular pyroptosis through the NLRP3/caspase-1/GSDMD pathway, and produces inflammatory mediators such as IL-1*β* and IL-18, triggering a series of inflammatory responses [16] that play important roles in the development of OA. Thus, in the present study, we explored the effects of four FAs on the TLR4/NF-*κ*B signaling pathway and the activation of the NLRP3 inflammasome and its downstream signals from the perspective of overall diet and single FAs. Our finding that diets rich in SFAs, MUFAs, or n-6 PUFAs increase the expression of the TLR4/NF-*κ*B signaling pathway and NLRP3 proteins, whereas a diet rich in n-3 PUFA did not significantly change protein expression, suggests that the pro- or anti-inflammatory effects of dietary FAs are related to the regulation of the TLR4/MyD88/NF-*κ*B signaling pathway and NLRP3 inflammasome.

We also observed that the expression levels of TLR4, NF-*κ*B, NLRP3, and IL-1*β* were not significantly elevated in joints from the n-3 PUFA group subjected to DMM compared with joints subjected to sham operation, consistent with the idea that the n-3 PUFA–rich diet had anti-inflammatory and antipyroptotic effects and contributed to postinjury repair. The cellular assays also showed that SFAs and n-6 PUFAs activated TLR4/NF-*κ*B and NLRP3/caspase-1/GSDMD signaling, whereas n-3 PUFAs had an inhibitory effect. The *in vitro* effects of MUFAs on these proteins differed from those observed in the animal experiment; MUFAs had anti-inflammatory and antipyroptotic effects *in vitro* that were similar to those of n-3 PUFAs.

SFA, acting as a non-microbial TLR4 agonist, can initiate TLR4-mediated inflammatory responses [37]. It can be recognized by the CD14-TLR4-MD2 complex and trigger inflammatory pathways, and may lead to alterations in the gut microbiota that produce excess LPS after high-fat intake, inducing a TLR4 inflammatory response through MyD88-dependent and/or MyD88-independent pathways, which in turn promote NF-*κ*B expression. PA has been shown to have pro-apoptotic and pro-inflammatory effects on articular cartilage through TLR4's upregulation of IL-6 and cyclooxygenase 2 expression in IL-1*β*–induced chondrocytes [49]. In addition, an SFA-enriched high-fat diet fed to mice activated NLRP3 inflammasomes in macrophages and dendritic cells and increased IL-1*β* secretion in adipose tissue [50], thereby promoting the progression of OA [51].

In contrast, n-3 PUFAs, such as DHA and eicosapentaenoic acid, exert anti-inflammatory effects by attenuating LPS or SFA activation of TLR4 signaling [52]. n-3 PUFAs can inhibit TLR4-induced signaling and target gene expression [53, 54], perhaps via anti-inflammatory effects mediated by G protein-coupled receptor 120. Oh et al. [55] found that the stimulation of G protein-coupled receptor 120 with n-3 PUFAs inhibited TLR4 signaling, possibly due to the binding of *β*-arrestin-2 to TAB1, leading to the inhibition of TAK1 phosphorylation and activation, which in turn blocks TLR4 signaling. In addition, n-3 PUFA treatment has been reported to disrupt TLR4 translocation to lipid rafts, thereby preventing TLR4 activation [53]. Additionally, Chen et al. [56] found that n-3 PUFA supplementation inhibited the expression of TLR4/NF-*κ*B signaling pathway–related proteins and reduced subsequent inflammatory responses. Similarly, n-3 PUFAs have been shown to inhibit NLRP3 inflammasome activity in human adipose tissue by down-regulating inflammatory gene expression in adipocytes and mononuclear macrophages [57]. Our study yielded similar results in mouse cartilage and SW1353 chondrocytes.

We found that the role of n-6 PUFA was similar to that of SFA and differed from that of n-3 PUFA. High n-6 PUFA intake has been reported to upregulate TLR4 expression [58], and a high dietary n-3/n-6 PUFA ratio has been shown to ameliorate obesity-associated inflammation and insulin resistance by inhibiting TLR4 activation in rats [58], which also reflects TLR4 upregulation by n-6 PUFA. An n-6 PUFA–enriched high-fat diet induced hepatic steatosis and fibrosis, along with the activation of the NLRP3 inflammasome, in mice [59], whereas the inhibition of n-6 PUFA metabolites reduced pro-inflammatory factor secretion and inhibited NLRP3 inflammasome activation [60]. Our results suggest that the effects of n-6 PUFA on TLR4 and the NLRP3 inflammasome are similar to those of SFA and that n-6 PUFA can promote OA progression.

The results of studies examining MUFA effects have been inconsistent. Some studies have shown that a MUFA-enriched high-fat diet stimulates the TLR4 inflammatory pathway by increasing JNK and STAT3 binding protein activation [61] and decreases NLRP3 inflammasome activation while reducing IL-1*β* secretion in adipose tissue and immune cells [50, 62]. OLA causes toxic damage to rat hepatocytes through the activation of MAPK/TLR4 signaling [63]. However, it has also been shown to inhibit TLR4-mediated inflammatory responses in monocytes and macrophages, reduce TLR4 and p–NF-*κ*B expression, and have anti-inflammatory effects [64]. In our animal experiments, we found that a MUFA-enriched high-fat diet increased the expression of TLR4/NF-*κ*B and NLRP3 inflammasome proteins, whereas chondrocytes treated with MUFA showed down-regulation of the TLR4/NF-*κ*B and NLRP3/caspase-1/GSDMD signaling pathways. This difference may reflect the ability of MUFA to inhibit TLR4 and NLRP3 inflammasome expression, with anti-inflammatory and anti-pyroptosis effects, but to a weaker extent than can n-3 PUFA. MUFA-rich high-fat feeds contain not only MUFAs but also other pro-inflammatory FAs, such as SFA and n-6 PUFA, which can weaken or even reverse the effects of MUFA, causing contradiction in the results of *in vitro* and *in vivo* experiments. Such contradictory results have been obtained in other studies. For example, Pietraszek et al. [65] found that MUFA-rich macadamia oil intake induced a postprandial anti-inflammatory response, but also increased the expression of pro-inflammatory genes, in healthy human subjects. Rocha et al. [66] showed that MUFA, although pro-inflammatory, had an anti-inflammatory effect relative to SFA-rich high-fat diets. These findings suggest that the effects of dietary MUFAs are influenced by various factors, including those related to the proportions of various FAs added to diets, feeding times, and tissue types.

Activation of the TLR4-mediated signaling pathway leads to NF-*κ*B p65 phosphorylation, which may be a key signal in NLRP3 inflammasome and pyroptosis activation [67, 68]. Yabuta and Shidoji [69] showed that the activation of the TLR4/NF-*κ*B signaling pathway activated NLRP3 inflammasomes, and thus caspase-1, generating GSDMD N-terminal fragments of the plasma membrane that induced cellular pyroptosis. To further clarify whether crosstalk between the TLR4/NF-*κ*B and NLRP3/caspase-1/GSDMD signaling pathways is part of the mechanisms underlying the actions of the four types of FA examined on chondrocytes in this study, we administered a TLR4 inhibitor to the SFA and n-6 PUFA groups and TLR4 overexpression lentivirus to the MUFA and n-3 PUFA groups, based on our preliminary data. When TLR4 was inhibited, the expression of the NLRP3 inflammasome and pyroptosis-related proteins caspase-1 and GSDMD was reduced after SFA or n-6 PUFA treatment, suggesting that the pro-inflammatory and pro-pyroptotic effects of SFA and n-6 PUFA are mediated through the TLR4/NF-*κ*B signaling pathway. When TLR4 was overexpressed, MUFA and n-3 PUFA supplementation reduced NLRP3, GSDMD, and caspase-1 expression, suggesting that the anti-inflammatory and anti-pyroptotic effects of MUFA and n-3 PUFA are also mediated through the TLR4 signaling pathway. Thus, TLR4/NF-*κ*B may play a more important role in mediating the effects of these four FAs on chondrocytes by acting as an upstream signal for the NLRP3/caspase-1/GSDMD signaling pathway.

## 5. Conclusions

**Figure 9:**
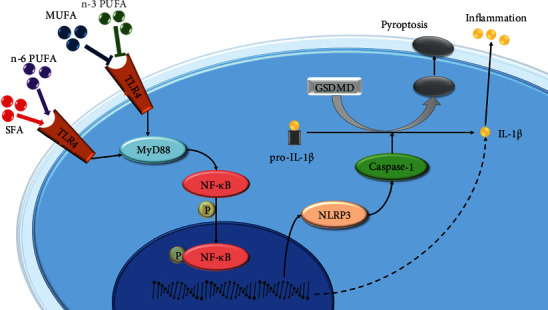
Schematic of the mechanism of FAs regulation of the NLRP3 inflammasome in chondrocytes.

In the present study, we found that SFA-, MUFA-, and n-6 PUFA–enriched diets were pro-inflammatory and pro-pyroptotic, promoting obesity-related OA, although the MUFA-enriched diet had a much weaker effect than did the other two diets. An n-3 PUFA–enriched diet had anti-inflammatory and anti-pyroptotic effects, protecting against obesity-related OA development. These effects are associated with activation/inhibition of the TLR4/NF-*κ*B signaling pathway in articular cartilage, which in turn upregulates/downregulates the NLRP3 inflammasome, thereby inducing cellular pyroptosis. Our results support the promotion of diets rich in n-3 PUFAs. MUFA may have anti-inflammatory and anti-pyroptotic effects, but the achievement of these effects may require adherence to appropriate FA ratios.  Figure 9 illustrates the mechanism of FA regulation of the NLRP3 inflammasome in chondrocytes. This study reveals possible mechanisms underlying the effects of different types of FA on OA. Additionally, our findings provide a theoretical basis for the prevention and treatment of obesity-related OA from a dietary perspective.

## Figures and Tables

**Table 1:** Relationships of biochemical and biomechanical factors to OA, determined by multiple regression.

Parameters	*β*	Sig.	95% C.I. for *β*
Biochemical			
FAs^a^	0.842	0.000	3.496-6.354
IL-1*β*	0.537	0.000	2.372-3.788
Biomechanical			
AUC_0-26wk_	—	—	—
AUC_19-26wk_	-0.229	0.065	-0.960-0.030

^a^Ordinal variable: LD and n‐3 = 0, SFA, MUFA, and n‐6 = 1. *β*: standardized coefficient.

## Data Availability

The data used to support the findings of this study are available from the corresponding author upon request.

## Supplementary Materials

Supplementary Materials Supplementary Table S1: composition of experimental diets. Supplementary Table S2: the standard of Modified Mankin score. Supplementary Table S3: OA cartilage histopathology grade assessment—OARSI. Supplementary Figure S1: transfection effect of TLR4 overexpressing lentivirus in chondrocytes.
