# Supplementary material for: Dietary Fatty Acid Regulation of the NLRP3 Inflammasome via the TLR4/NF-κB Signaling Pathway Affects Chondrocyte Pyroptosis
Source: Oxid Med Cell Longev. 2022 May 4;2022:3711371. doi: 10.1155/2022/3711371 (PMC9095358; doi:10.1155/2022/3711371)
Supplement: Supplementary Materials — Supplementary Table S1: composition of experimental diets. Supplementary Table S2: the standard of Modified Mankin score. Supplementary Table S3: OA cartilage histopathology grade assessment—OARSI. Supplementary Figure S1: transfection effect of TLR4 overexpressing lentivirus in chondrocytes. [file 3711371.f1.docx]

Table S1: Composition of experimental diets.

|  | **Low-fat diet** | | **High-fat diet** | | | | | | | |
| --- | --- | --- | --- | --- | --- | --- | --- | --- | --- | --- |
|  | LD | | SFA | | MUFA | | n-6 PUFA | | n-3 PUFA | |
| **Content** | g | kcal% | g | kcal% | g | kcal% | g | kcal% | g | kcal% |
| Protein | 19.2 | 20 | 26.2 | 20 | 26.2 | 20 | 26.2 | 20 | 26.2 | 20 |
| Carbohydrate | 67.3 | 70 | 26.3 | 20 | 26.3 | 20 | 26.3 | 20 | 26.3 | 20 |
| Fat | 4.3 | 10 | 34.9 | 60 | 34.9 | 60 | 34.9 | 60 | 34.9 | 60 |
| Total | 90.8 | 100 | 87.4 | 100 | 87.4 | 100 | 87.4 | 100 | 87.4 | 100 |
| kcal/g |  | 3.85 |  | 5.24 |  | 5.24 |  | 5.24 |  | 5.24 |
|  |  |  |  |  |  |  |  |  |  |  |
| **Ingredient** | g | kcal | g | kcal | g | kcal | g | kcal | g | kcal |
| Casein | 200 | 800 | 200 | 800 | 200 | 800 | 200 | 800 | 200 | 800 |
| L-Cystine | 3 | 12 | 3 | 12 | 3 | 12 | 3 | 12 | 3 | 12 |
| Corn starch | 315 | 1260 | 0 | 0 | 0 | 0 | 0 | 0 | 0 | 0 |
| Maltodextrin | 35 | 140 | 125 | 500 | 125 | 500 | 125 | 500 | 125 | 500 |
| Sucrose | 350 | 1400 | 68.8 | 275 | 68.8 | 275 | 68.8 | 275 | 68.8 | 275 |
| Cellulose | 50 | 0 | 50 | 0 | 50 | 0 | 50 | 0 | 50 | 0 |
| Soybean oil | 25 | 225 | 120 | 1080 | 140 | 1260 | 40 | 360 | 150 | 1350 |
| Lard | 20 | 180 | 100 | 900 | 70 | 630 | 60 | 540 | 90 | 810 |
| Corn oil |  |  |  |  |  |  | 170 | 1530 |  |  |
| Olive oil |  |  |  |  | 60 | 540 |  |  |  |  |
| Fish oil |  |  |  |  |  |  |  |  | 30 | 270 |
| Cocoanut oil |  |  | 50 | 450 |  |  |  |  |  |  |
| Vitamin Mix | 10 | 40 | 10 | 40 | 10 | 40 | 10 | 40 | 10 | 40 |
| Mineral Mix | 10 | 0 | 10 | 0 | 10 | 0 | 10 | 0 | 10 | 0 |
| Dicalcium phosphate | 13 | 0 | 13 | 0 | 13 | 0 | 13 | 0 | 13 | 0 |
| Calcium carbonate | 5.5 | 0 | 5.5 | 0 | 5.5 | 0 | 5.5 | 0 | 5.5 | 0 |
| Potassium citrate | 16.5 | 0 | 16.5 | 0 | 16.5 | 0 | 16.5 | 0 | 16.5 | 0 |
| Choline bitartrate | 2 | 0 | 2 | 0 | 2 | 0 | 2 | 0 | 2 | 0 |
| Total | 1055 | 4057 | 773.8 | 4057 | 773.8 | 4057 | 773.8 | 4057 | 773.8 | 4057 |
|  |  |  |  |  |  |  |  |  |  | |
| SFA (% of kcal) | 2.6 |  | 23.5 |  | 13.0 |  | 11.6 |  | 14.8 |  |
| MUFA (% of kcal) | 3.3 |  | 16.8 |  | 24.1 |  | 18.2 |  | 19.4 |  |
| PUFA (% of kcal) | 3.8 |  | 18.5 |  | 22.0 |  | 29.3 |  | 24.6 |  |
|  |  |  |  |  |  |  |  |  |  |  |
| n-6 | 15.1 |  | 73.5 |  | 87.2 |  | 126.7 |  | 89.7 |  |
| n-3 | 2.0 |  | 9.9 |  | 11.8 |  | 5.2 |  | 21.3 |  |
| n-6:n-3 ratio | 7.4 |  | 7.4 |  | 7.4 |  | 24.6 |  | 4.2 |  |

Table S2: The standard of Modified Mankin score.

| **Parameters** | **Grade** |
| --- | --- |
| **Pericellular Safranin O staining** |  |
| Normal | 0 |
| Slightly enhanced | 1 |
| Intensely enhanced | 2 |
|  |  |
| **Background Safranin O staining** |  |
| Normal | 0 |
| Slight increase or decrease | 1 |
| Severe increase or decrease | 2 |
| No staining | 3 |
|  |  |
| **Arrangement of chondrocytes** |  |
| Normal | 0 |
| Appearance of clustering | 1 |
| Hypocellularity | 2 |
|  |  |
| **Cartilage structure** |  |
| Normal | 0 |
| Fibrillation in the superficial layer | 1 |
| Fibrillation beyond the superficial layer | 2 |
| Missing articular cartilage | 3 |

Table S3: OA cartilage histopathology grade assessment—OARSI.

| Grade (key feature) | Associated criteria (tissue reaction) |
| --- | --- |
| Grade 0: surface intact, cartilage morphology intact | Matrix: normal architecture |
|  | Cells: intact, appropriate orientation |
|  |  |
| Grade 2: surface discontinuity | As above |
|  | + Matrix discontinuity at superficial zone (deep fibrillation) |
|  | ± Cationic stain matrix depletion (Safranin O or Toluidine Blue) upper 1/3 of cartilage |
|  | ± Focal perichondronal increased stain (mid zone) |
|  | ± Disorientation of chondron columns |
|  | Cells: death, proliferation (clusters), hypertrophy |
|  |  |
| Grade 3: vertical fissures (clefts) | As above |
|  | Matrix vertical fissures into mid zone, branched fissures |
|  | ± Cationic stain depletion (Safranin O or Toluidine Blue) into lower 2/3 of cartilage (deep zone) |
|  | ± New collagen formation (polarized light microscopy, Picro Sirius Red stain) |
|  | Cells: death, regeneration (clusters), hypertrophy, cartilage domains adjacent to fissures |
|  |  |
| Grade 4: erosion | Cartilage matrix loss: delamination of superficial layer, mid layer cyst formation |
|  | Excavation: matrix loss superficial layer and mid zone |
|  |  |
| Grade 5: denudation | Surface: sclerotic bone or reparative tissue including fibrocartilage within denuded surface. Microfracture with repair limited to bone surface |
|  |  |
| Grade 6: deformation | Bone remodelling (more than osteophyte formation only). Includes: microfracture with fibrocartilaginous and osseous repair extending above the previous surface |


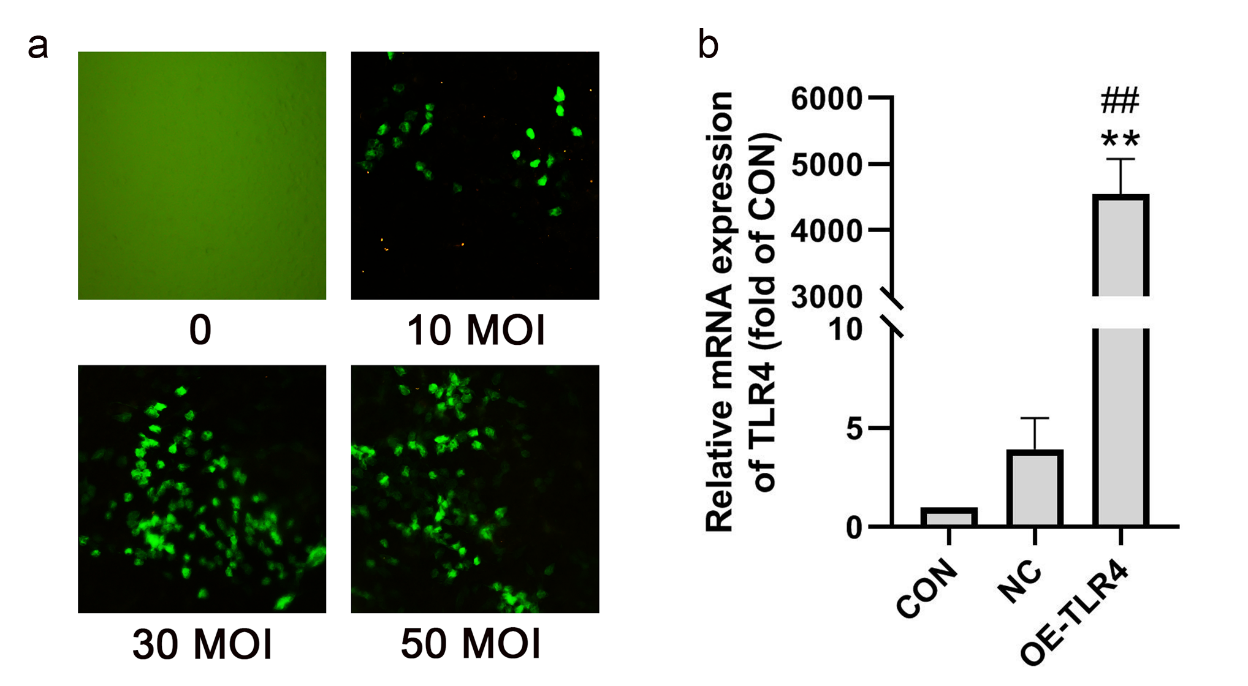
Figure S1: Transfection effect of TLR4 overexpressing lentivirus in chondrocytes. (a) Chondrocytes were transfected with 10MOI, 30MOI and 50MOI TLR4 lentivirus for 72 h, respectively. Cells expressing green fluorescently labeled protein were successfully transfected, and the transfection efficiency of 30MOI and 50MOI was similar. (b) The expressions of TLR4 were measured using RT-PCR. Compared with CON and NC groups, the expression level of TLR4 mRNA in 30MOI of transfected cells was significantly increased.
